# Investigating the Size and Microstrain Influence in the Magnetic Order/Disorder State of GdCu_2_ Nanoparticles

**DOI:** 10.3390/nano10061117

**Published:** 2020-06-05

**Authors:** E. M. Jefremovas, J. Alonso, M. de la Fuente Rodríguez, J. Rodríguez Fernández, J. I. Espeso, D. P. Rojas, A. García-Prieto, M. L. Fernández-Gubieda, L. Fernández Barquín

**Affiliations:** 1 Dpto. CITIMAC, Facultad de Ciencias, Universidad de Cantabria, 39005 Santander, Spain; javier.alonsomasa@unican.es (J.A.); maria.delafuente@unican.es (M.d.l.F.R.); jesus.rodriguez@unican.es (J.R.F.); jose.espeso@unican.es (J.I.E.); barquinl@unican.es (L.F.B.); 2 Dpto. Estructuras y Física de la Edificación, ETSAM, Universidad Politécnica de Madrid, 28040 Madrid, Spain; d.rojas@upm.es; 3 Dpto. de Física Aplicada I, Escuela de Ingeniería de Bilbao, 48013 Bilbao, Spain; ana.garciap@ehu.eus; 4 Dpto. de Electricidad y Electrónica, Universidad del País Vasco—UPV/EHU, 48940 Leioa, Spain; malu.gubieda@ehu.eus

**Keywords:** magnetic nanoparticles, rare earth intermetallics, magnetic coupling, X-ray diffraction, Spin Glass

## Abstract

A series of GdCu${}_{2}$ nanoparticles with controlled sizes ranging from 7 nm to 40 nm has been produced via high-energy inert-gas ball milling. Rietveld refinements on the X-ray diffraction measurements ensure that the bulk crystalline Imma structure is retained within the nanoparticles, thanks to the employed low milling times ranging from *t* = 0.5 to *t* = 5 h. The analysis of the magnetic measurements shows a crossover from Superantiferromagnetism (SAF) to a Super Spin Glass state as the size decreases at NP size of 〈D〉≈ 18 nm. The microstrain contribution, which is always kept below 1%, together with the increasing surface-to-core ratio of the magnetic moments, trigger the magnetic disorder. Additionally, an extra contribution to the magnetic disorder is revealed within the SAF state, as the oscillating RKKY indirect exchange achieves to couple with the aforementioned contribution that emerges from the size reduction. The combination of both sources of disorder leads to a maximised frustration for 〈D〉≈ 25 nm sized NPs.

## 1. Introduction

Rare Earth (R) intermetallic alloys constitute excellent systems for studying the 4*f*-orbital magnetism, the eventual effect of crystal field (CEF) and the indirect exchange RKKY interactions that develop within these systems [1]. Usually, their high magnetic moment is a handy ingredient for their technological applications as permanent magnets [2,3] or giant magnetostriction [4] alloys. In the very recent years, magnetic nanoparticles (MNPs) containing Rare Earths in their composition have been proposed as promising candidates in applications mainly connected to magnetic separation and biomedicine [5,6]. Among these MNPs, Gd-based ones are especially interesting for biomedical applications, e.g., as contrast agents for MRI imaging [7,8]. For this kind of applications, a profound understanding of the magnetic properties of Rare Earths in nanoparticle form is mandatory. Nevertheless, it is surprising that the outburst of magnetic Nanoscience at the turn of the century has not paid much attention to the magnetism at its basis in 4*f*-based compounds, in general, and to the intermetallics in particular. It has only been very recently, around a decade ago, that some works started to unveil the magnetic properties of *R*-intermetallics at the nanoscale in the form of collections of magnetic nanoparticles (NPs) [9,10,11]. These works showed how the bulk magnetic features do remain within the nanoscale, but in a different fashion. Moreover, these results also underlined the importance of carefully defining the nanometric structure to understand whatever nanomaterial is analysed.

Bulk intermetallic alloys are usually formed by a binary or ternary combination of elements. These normally include a magnetic *R*-ion that couples magnetically in an oscillatory Ferro (FM) or Antiferromagnetic (AF) fashion over long-ranged distances via the conduction electrons, i.e., RKKY indirect exchange [12]. The distance between *R*-ions can be adjusted by the crystallographic structure, which is therefore a driving ingredient to either modify the intensity and/or the sign of the magnetic coupling. Consequently, a natural way to modify this distance is by reducing the dimensionality of the bulk material, i.e., by milling the master (bulk) alloy.

One of the initial examples of milled 4*f*-intermetallic alloys was GdAl${}_{2}$ [13,14], which was classified as a re-entrant spin glass, showing FM and spin glass (SG) transitions. Interestingly, $Gd^{3+}$ is a *S*-state ion (*L* = 0) and, in consequence, no spin-orbit interaction is present, resulting in zero intrinsic magnetocrystalline anisotropy [15]. Thus, the only type of magnetic anisotropy for Gd-intermetallic compounds is the one coming from the two-ion interactions [16]. Instead of FM order, as in other Gd-based intermetallics, bulk GdCu${}_{2}$ alloys have shown AF coupling [17,18]. Nevertheless, there are no reported works on the evolution of the magnetic properties when turning this alloy into the nanoscale. In general, the literature is again very scarce for RCu${}_{2}$ intermetallic NPs [19,20]. A recent example is the work on TbCu${}_{2}$ NPs [21,22], as Tb${}^{3+}$-ions are the most similar to Gd${}^{3+}$-ones in terms of *de Gennes* factor and $T{}_{N}$ [15]. The aforementioned works have shown how the AF state survives within the mesoscopic scale together with the emergence of a disorder contribution in the form of a Spin Glass (SG) state, forming the so-called Superantiferromagnetic (SAF) state [23]. Hence, the issue that remains open is to precisely determine the impact that size reduction to the nanoscale has in CEF-free alloys with RKKY interactions.

For this purpose, we present here a structural and magnetic analysis for six alloys in a series of GdCu${}_{2}$ NPs of different sizes. The study shows how the size reduction within the nanoscale tunes the RKKY interactions among the magnetic moments. In this way, the GdCu${}_{2}$ NP system evolves from a SAF state, similar to the one of TbCu${}_{2}$ NPs, to a completely disordered but still interacting state of Super Spin Glass (SSG), which is not found in the aforementioned TbCu${}_{2}$ system [21]. As the studied GdCu${}_{2}$ NPs retain the bulk Imma crystalline structure, the downsizing and the microstrain are the ingredients responsible for the destruction of long range magnetic order. Additionally, in order to characterise the stability of the Spin Glass phase with respect to the size, two novel quantities have been analysed: the area between Zero Field Cooling-Field Cooling (ZFC-FC) magnetisation branches (named as Irreversibility Area, IA) and the Full Width at Half Maximum (FWHM) of the SG ZFC-M(H,T) cusp (defined as Spin Glass sharpness FP). These two quantities point to NPs of 〈D〉 = 25(5) nm to be the ones where the Spin Glass phase is maximum. This finding, that is in contrast with the intuitive idea of increasing frustration with decreasing size [11,24], constitutes the main novelty in this study of AF (RKKY coupled) NPs.

## 2. Materials and Methods

Polycrystalline pellets of GdCu${}_{2}$ were obtained in an arc furnace (MAM-1 Johanna Otto Gmbh, Germany) under an Ar atmosphere (99.99%) from stoichiometric amounts of Gd (99.9%) and Cu (99.999%) pure metals, obtaining large (∼5 g) quantities of alloy. The reduction to the nanoscale was achieved in a high-energy planetary ball mill with carbide-tungsten (WC) air-tight containers (Retsch PM 400/2, Germany) sealed off under Ar atmosphere (99.99%) to minimise the presence of oxygen. Nevertheless, if at all, this oxide is present, it is only concerned to no more than a surface layer, and its low ordering temperature ($T_{N}$∼ 18 K [25] for Gd${}_{2}$O${}_{3}$ and $T_{N}$ below 13 K [26] in Gd${}_{2}$O${}_{3}$ NPs) minimises the influence in transitions found at higher temperatures. A total of six series of alloys were produced and milled for times *t* = 0.5, 1, 1.5, 1.75, 2 and 5 h in order to obtain six different series of NPs of different sizes.

X-ray diffraction (XRD) measurements were performed at room temperature in a Bruker D8 Advance diffractometer (Germany) working in Bragg-Bentano geometry, and using Cu-Kα (λ = 1.5418 Å) radiation. The selected range for 2θ Bragg angle was 18${}^{{}^{\circ}}$ to 95${}^{{}^{\circ}}$, with an angular step of 0.02${}^{{}^{\circ}}$ and 1 s/step. A high count rate Lynxeye detector is mounted in the X-ray diffactometer.

DC and AC magnetic susceptibility measurements were performed in both Quantum Design QD-PPMS and QD-MPMS (SQUID) magnetometers (CA, USA) in the temperature range of *T* = 5–300 K for most measurements. In some special cases (e.g., checking out the exchange bias effect), the minimum temperature was *T* = 2 K. For the static DC magnetization ($M{}_{DC}$), magnetic fields $\mu_{0}H$≤ 5 T have been used for the nanoscaled samples, whereas for the bulk alloy the magnetization was recorded up to $\mu_{0}H$ = 9 T. AC-susceptibility ($\chi_{AC}$) data were obtained with an oscillating field of *h* = 0.313 mT and frequencies (*f*) ranging from 0.01 kHz to 10 kHz in the case of the QD-PPMS measured ones, and with *h* = 0.1 mT and *f* = 0.2–300 Hz for the ones recorded in the QD-MPMS.

## 3. Results and Discussion

### 3.1. Structural Characterisation

**Figure 1:**
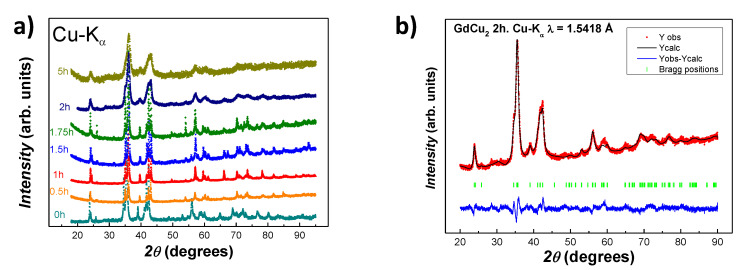
(**a**) XRD profile for GdCu${}_{2}$ samples. Patterns with increasing milling time have been shifted up and re-scaled for clarity. (**b**) XRD pattern (red), Rietveld refinement (black) and Bragg positions for the hkl peaks (green) obtained for GdCu${}_{2}$ 2h milled NPs. The blue line below the spectrum represents the difference between the experimental and the calculated patterns.

XRD patterns corresponding to the bulk alloy (*t* = 0 h) and the milled GdCu${}_{2}$ powders (t=0.5,1,1.5,1.75,2 and 5 h) are shown in Figure 1a. The patterns display the presence of peaks corresponding to an orthorhombic Imma structure, with a progressive peak broadening. This is associated with both the size reduction and the appearance of a microstrain η introduced by the milling process [27,28]. The later microstrain contribution slightly distorts the crystalline array of ions, but previous works [21,29] have shown that for milling times t≤ 5 h, the crystallinity of the samples is well preserved.

All XRD patterns have been refined employing the Rietveld method with the Fullprof suite [30]. We have used a Thompson-Cox-Hasting analysis that allows us to determine both the mean NP size, 〈D〉 and the microstrain η. Briefly, the broadening of the diffraction peaks is the result of three contributions: (i) one dependent on the cos(θ) function (Scherrer formula), which provides information on the 〈D〉; (ii) a second one, that follows a tan(θ) angular dependence, which gives information on the η; and (iii) the instrumental function, extracted from a calibration using a standard LaB${}_{6}$ sample. Further details can be found in [31]. As an example of the Rietveld calculations, Figure 1b shows the fitting performed for *t* = 2 h GdCu${}_{2}$ milled NPs. The main structural parameters for all of the milled alloys are summarised in Table 1. From these values, one can notice that the unit cell experiences a slight expansion when nanoscaled, that can be attributed to a relaxation of the unit cell [28]. Such expansions ΔV are below 1%. These results indicate that the bulk orthorhombic CeCu${}_{2}$-type crystal structure (Imma space group) is maintained within the core of all the NP sizes analysed in this study. Furthermore, the unit cell parameters are in good agreement with what has been previously reported for bulk GdCu${}_{2}$ alloy [15]. Regarding the values for the microstrain, these are always kept below 1%, according to previous works [10,11].

**Figure 2:**
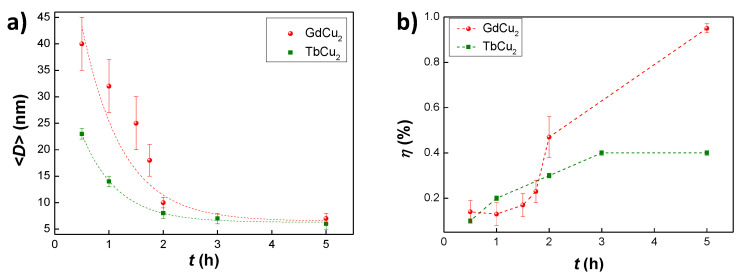
Evolution of the (**a**) mean diameter size 〈D〉 and (**b**) microstrain η of GdCu${}_{2}$ NPs (red spheres) and TbCu${}_{2}$ (green squares) (data for the latter taken from [20]) with milling time *t*. Dashed lines are sketched as a guide for the eyes.

The average NP sizes are also shown in Table 1. The first striking result is that, already for very short milling times (*t* = 0.5 h) nanometric sizes are reached (〈D〉∼40 nm). This is in contrast with the long milling times t≥ 20 h usually employed in GdX${}_{2}$ nano alloys (where *X* is a 3*d* metal) [32]. Secondly, the evolution of size with milling time plotted in Figure 2a follows a similar reduction as the one found in other RCu${}_{2}$ nano-alloys (for instance TbCu${}_{2}$ [21], also plotted for comparison). However, the decrease of size is more drastic than in TbCu${}_{2}$ for low milling times, reaching almost a saturation in size reduction for milling times beyond t≥ 2 h. On the other hand, the microstrain for GdCu${}_{2}$ nanoparticles follows a different behaviour than in TbCu${}_{2}$ (Figure 2b). The microstrain in GdCu${}_{2}$ NPs rises fast for t≥ 2 h, whereas in TbCu${}_{2}$ is almost saturated by then. As the size is nearly the same for the two alloys at *t* = 5 h, this higher η for GdCu${}_{2}$ NPs would indicate an increased presence of defects in the particle core and shell with respect to the TbCu${}_{2}$ case.

### 3.2. Magnetic Characterisation

#### 3.2.1. Static Magnetic Susceptibility vs. Temperature

**Figure 3:**
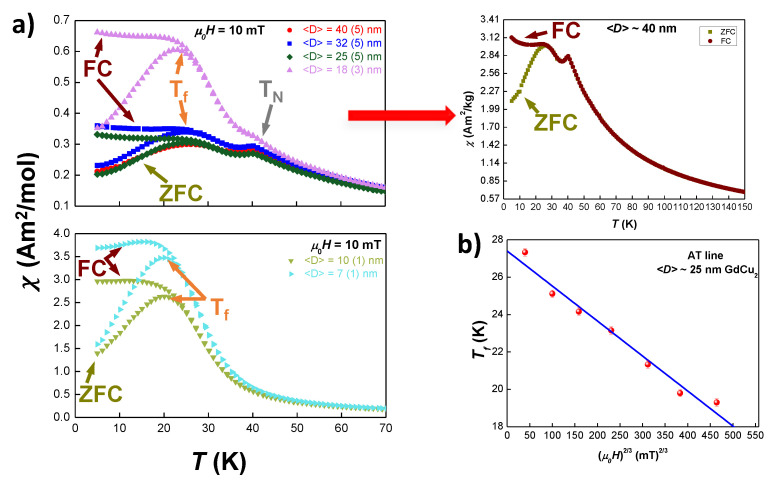
(**a**) ZFC (marked with dark yellow arrows) and FC (brown arrows) curves of DC-susceptibility of the milled samples, at $\mu_{0}H$ = 10 mT. Top: GdCu${}_{2}$ NPs where $T_{N}$ and $T_{f}$ coexist (i.e., 〈D〉≥ 18 nm). Measurements for the data corresponding to the larger NPs (〈D〉= 40(5) nm) are represented individually at the right side, where different colours have been employed to distinguish between the ZFC branch (dark yellow) and the FC one (brown). Bottom: GdCu${}_{2}$ NPs where the $T_{N}$ transition is suppressed (〈D〉≤ 10 nm). Both plots reveal an increasing $\chi_{DC}$ when reducing the NP size. (**b**) Variation of the freezing temperature $T_{f}$ versus H${}^{2/3}$ for 〈D〉≈ 25 nm GdCu${}_{2}$ NPs.

Zero-Field Cooled (ZFC) and Field Cooled (FC) measurements have been performed at fields $\mu_{0}H$ = 2.5–100 mT for all the nano-scaled GdCu${}_{2}$ alloys. In Figure 3a, ZFC (pointed with a dark yellow arrow)-FC (brown arrow) curves for $\mu_{0}H$ = 10 mT have been plotted for all samples. For a more clear depiction of the differences in the magnetic behaviour, we have split the data in two sets of curves: 〈D〉≥ 18 nm (i.e., milling times t< 2 h), and 〈D〉≤ 10 nm (t≥ 2 h). Additionally, we have included separately the ZFC-FC measurements for 〈D〉= 40(5) nm (t= 0.5 h) NPs, as they overlap with the ones corresponding to 〈D〉= 32(5) nm. All of the data sets exhibit magnetic irreversibility, which is the separation between the ZFC and FC curves, but there are some subtle differences that deserve a closer inspection.

In the low milling time regime, t≥ 1.75 h, i.e., NPs with 〈D〉≥ 18 nm (Figure 3a top panel), two peaks can be observed: the first one, located around $T_{N}=40.2$ K, that corresponds to the Néel temperature of the bulk alloy [17], and a second one, peaking around T∼25 K. The latter is associated with a freezing transition to a Spin Glass (SG) phase. This SG state cannot be considered a canonical phase and often this behaviour is named as Spin Glass-like phase. The double transition reveals the re-entrant magnetic behaviour of these NPs, and is similar to the one described for TbCu${}_{2}$ NPs in [21]. Considering the results obtained in that work, these two transitions in the ZFC-FC curves would indicate that the GdCu${}_{2}$ NPs are in the so-called SAF state, where the magnetic moments located within the core retain the AF coupling order, while the ones situated at the shell display a disordered SG-like state.

Regarding the Néel temperature, we can observe that there is no size dependence, as no shift of this transition is measured within the range 〈D 〉∼[18, 40] nm (t≥ 1.75 h). This finding is striking, as a shift of $T_{N}$ to lower temperatures with the size reduction has been reported in some recent works. This shift has been attributed to either the way that surface spin couple [33] or finite size effects [21] as it happened in TbCu${}_{2}$ NPs. Following this work on TbCu${}_{2}$ NPs, and taking into account the different unit cell distortions, we would expect to observe a relative shift of $\Delta V(TbCu_{2})\cdot \Delta T_{N}\left(D\right)\left(TbCu_{2}\right)∼\frac{0.11}{0.36}\cdot \left(-0.11\right)∼-0.034$ for GdCu${}_{2}$ NPs. This would imply an absolute shift of $\Delta T_{N}\left(18nm\right)≃-1.4K$, a value that is large enough to be detected in standard $M{}_{DC}\left(T\right)$ measurements. Hence, the absence of shift in T${}_{N}$ implies that the unit cell distortion is not enough to modify the magnetic coupling among Gd atoms within the core of the NPs for 〈D〉≥ 18 nm.

By contrast, the survival of the AF interactions shows a clear size dependence. As it can be observed from Figure 3a bottom panel, the intensity of the AF peak decreases with milling time, being completely wiped out for 〈D〉≈ 10 nm (t= 2 h). The progressive decrease of this peak is related to the increasing number of disordered Gd-moments located in the shell. In this way, as the NP size decreases, both the shell-to-core ratio of magnetic moments and the microstrain increase. These two contributions favour the magnetic disorder, in opposition to the AF order coupling, which is constrained to the NPs core. Thus, in GdCu${}_{2}$-NPs, there is a critical size (D≤ 10 nm) below which the AF peak completely disappears and the contribution to the magnetic response of the NPs is mostly due to those disordered moments. For $T>T{}_{N}$, both ZFC and FC branches overlap, which further evidences the AF character of this transition [34].

On the other hand, the behaviour of the peak associated with the SG-like phase displays some uncommon features. Although the intensity of the peak increases as milling time does, this uprise is far from being constant, as it was found for other RX${}_{2}$ NPs [20,21]. In Figure 3a top panel, it can be observed how, as long as AF ordering is still present, the increase of the intensity of the peak associated with $T_{f}$ is small (less than 10%). However, for NPs 〈D〉≤ 25 nm (i.e., t≥ 1.5 h), the peak amplitude greatly increases: from 〈D〉≈ 25 nm (t= 1.5 h) to 〈D〉≈ 18 nm (t= 1.75 h) it doubles but, from 〈D〉≈ 18 nm to 〈D〉≈ 10 nm (t= 2 h), $\chi^{\prime }\left(H,T\right)$ is multiplied by 4. Then, the increase found for smaller NP sizes (from 〈D〉≈ 10 nm to 〈D〉≈ 7 nm) stabilises, being only 25%. The inspection of the FC branch further supports the SG nature of the low temperature transition, as the *plateau* shape expected for SG systems for $T<T_{f}$ is recovered [17,35].

In order to further corroborate the SG character of this transition, the behaviour of the $T_{f}$ with an external applied field $\mu_{0}H$ has been scrutinised. The results follow an de Almeida-Thouless (AT) line [36,37] (see Figure 3b for GdCu${}_{2}$ NPs of 〈D〉≈ 25 nm as an example):

(1) $$H\left(T\right)\propto {\left(1-\frac{T{}_{f}\left(H\right)}{T{}_{f}\left(0\right)}\right)}^{m}$$

where *m* is 3/2, within the mean field framework for Spin Glasses [36,37]. However, there is an exception to this general behaviour in the case of NPs with 〈D〉≈ 40 nm (i.e., *t* = 0.5 h). This disagreement is clearly due to the remarkable presence of AF order with respect to the ill-defined SG phase at this 〈D〉. In addition to the fitting, the extrapolation of the AT-line to $\mu_{0}H$ = 0 T gives a valuable estimation of $T{}_{irrev}$ ($\mu_{0}H$ = 0), where a true SG is established [35]. Later on, the value for this estimated $T_{irrev}\left(\mu_{0}H=0\right)$ will be compared with the value of $T_{f,0}$ obtained from $\chi {}_{AC}\left(T,f\right)$ measurements. We will now just mention that both values differ in only 2 K, a difference that is in good agreement with the literature [37].

All in all, with respect to the results of $M{}_{DC}\left(H,T\right)$ in ZFC-FC sequences, two regimes can be established: one, for GdCu${}_{2}$ NPs with sizes above 〈D〉≈ 18 nm, in which there is a coexistence of AF + SG, where the AF interactions lock the magnetic moments in an ordered state, tending to prevent them to get into the disordered SG-like phase; and a second one, for NPs with sizes below 〈D〉≈ 10 nm, in which only a SG state is established (thus, in a so-called Super Spin Glass state, SSG), becoming favoured by the AF suppression, concomitant to the size reduction.

In order get more information, a Curie-Weiss fit (1/*χ* vs. *T* for $T>T{}_{N}$ at $\mu_{0}H$ = 0.1 T) has been performed. The results for the obtained Curie Temperature $\theta {}_{P}$ and effective magnetic moment $\mu_{eff}$ are displayed in Table 2. Interestingly, the positive value of $\theta {}_{P}$ points to the existence of FM interactions among magnetic moments [34]. It is worth noting that the $\theta {}_{P}$ values stay almost constant with size reduction down to 〈D〉≈ 25 nm (t= 1.5 h), while the AF interactions are still relevant. The obtained $\theta_{P}∼$ 8 K are close to the the bulk value of $\theta_{P}^{bulk}$ = 8.05(2) K (which is slightly higher than the reported 7 K [38]). Once the size of NPs is further reduced from 〈D〉≈ 25 nm, the $\theta {}_{P}$ value starts to increase. In this way, from 〈D〉≈ 25 nm to 〈D〉≈ 18 nm, there is a moderate change (25 %) but then, when the 〈D〉≈ 10 nm (i.e, the limiting size for the AF arrangement survival is overcome), $\theta {}_{P}$ gets doubled.

Regarding $\mu_{eff}$ values, these remain almost constant with the size reduction. This is expected, as only the Gd${}^{3+}$-ions are responsible for the magnetism. It is worth mentioning that the values are slightly higher than those expected theoretically, as $\mu_{eff}=g_{J}$$\mu_{B}$$\sqrt{J(J+1)}=$ 7.94 $\mu_{B}$, where *J* = 7/2 for $Gd^{3+}$ ions. This higher experimental moment has also been reported in single crystal [39] ($\mu_{eff}$ = 8.14 $\mu_{B}$) and polycrystalline [40] ($\mu_{eff}$ = 8.7 $\mu_{B}$) GdCu${}_{2}$ bulk alloys, and has been attributed to conduction-electron enhancement effects for ferromagnetic GdAl${}_{2}$ [41].

We will now discuss two novel (and simple) quantities that have been introduced in this work in order to get a better insight about the robustness of the Spin Glass state. These are (i) the area between ZFC-FC magnetisation branches (named as Irreversibility Area, IA) and (ii) the Full Width at Half Maximum (FWHM) of the SG ZFC-M(H,T) cusp (defined as Spin Glass sharpness FP). To obtain the IA, ZFC and FC M${}_{DC}$ values are subtracted and normalised to the maximum FC $M{}_{DC}$ value. For the FP, the FWHM is measured from the normalised $M/M^{max}$ vs. $T/T{}_{f}$ curves. Both quantities are dimensionless. The value of IA tends to increase when the presence of the SG phase in the NPs ensemble is strong. On the other hand, the FP is related to the collective freezing of moments: the faster they freeze, the sharper the peak is (thus, the smaller the FP). In short, higher values for IA, and smaller for FP, would indicate greater robustness of the SG state.

**Figure 4:**
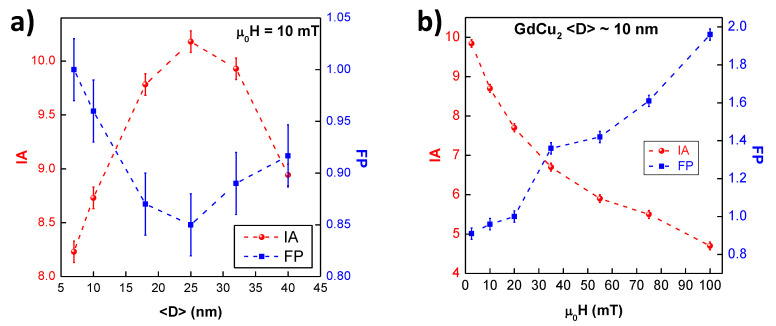
Evolution of the Irreversibility Area IA (red spheres) and the Spin Glass sharpness FP (blue squares) with respect to (**a**) mean diameter size for the different GdCu${}_{2}$ NPs (the latter without error bars for clarity) and (**b**) $\mu_{0}H$ for 10 nm-sized GdCu${}_{2}$ NPs (2 h milled).

Values for both IA and FP with respect to the NP size are shown in Figure 4a. One would expect that, with decreasing size (increasing microstrain), the disorder would be enhanced, thus, a progressive increase in the IA values and decrease in the FP values should be observed. However, a maximum (minimum) occurs for the IA (FP) when the size is D≅ 25 nm. This non-progressive behaviour can be understood taking into account that, besides the disorder introduced by the size reduction (increasing shell-to-core ratio and microstrain), there is a competition between FM and AF interactions that leads to an enhanced frustration. This idea of exchange order interactions helping disorder SG phases has already been discussed for FM order in [42]. It seems that, as long as AF interactions remain within our NPs, two spin networks are established: one mostly corresponding to the spins in the core, with competing FM-AF RKKY interactions; and another one, mainly related to the spins in the shell, where the increasing microstrain introduced by the milling gives rise to a higher magnetic disorder. Figure 4b shows the evolution of IA and FP as a function of the $\mu_{0}H$ for GdCu${}_{2}$〈D〉≈ 10 nm NPs (t= 2 h). For these smaller NPs (i.e., larger milling times) the magnetic disorder is prevalent, and, consequently, the ensemble of MNPs should be labelled as a Super Spin Glass, SSG [24,43]. It can be observed that, as the magnetic field is increased, the IA is reduced, and, at the same time, FP increases, indicating the progressive destruction of SG state with increasing magnetic field, as found in [11].

#### 3.2.2. Isothermal Magnetic Susceptibility

**Figure 5:**
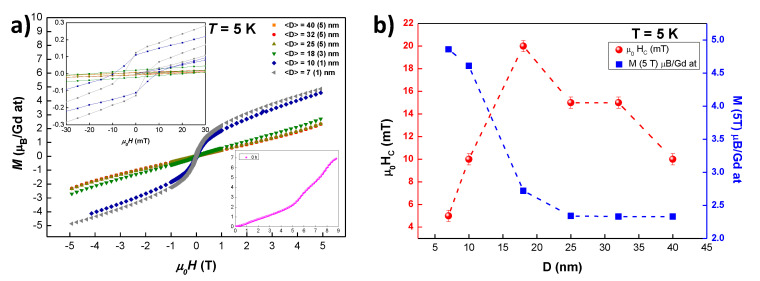
(**a**) Magnetic moment per Gd atom *M* ($\mu {}_{B}$/ Gd at) vs. external applied field $\mu_{0}H$ (hysteresis loop) measured at *T* = 5 K for the GdCu${}_{2}$ NPs. Left inset: Zoom of the central region $\mu_{0}H∼$ mT to better see the coercive field $\mu_{0}H{}_{C}$ and remanent magnetisation $M{}_{r}$. Right inset: $M(\mu_{0}H)$ for the bulk (*t* = 0 h) alloy measured up to $\mu_{0}H$ = 9 T. (**b**) Evolution of the $\mu_{0}H{}_{C}$ (red spheres) and *M* (5T) (blue squares) with the mean diameter 〈D〉 of the NPs measured at *T* = 5 K.

$M{}_{DC}(\mu_{0}H,T=5$ K) loops are shown in Figure 5a. In the range of fields employed, none of the alloys reaches the magnetic saturation. This can be related to the large anisotropy contribution due to the canting of magnetic moments at the surface of the NPs [21]. Noticeably, an abrupt increase of $M{}_{DC}$ ($\mu_{0}H$ = 5 T; *T* = 5 K) has been found when the AF arrangement is removed (i.e., 〈D〉≤ 10 nm; t≥ 2 h). This rise in the M(H) magnitude is understood in terms of the increase of the FM couplings that are established among some magnetic moments (see $\theta {}_{P}$ values in the previous section). The destruction of the AF arrangement triggers a change in the shape of the *M* vs. $\mu_{0}H$ curve when 〈D〉≤ 10 nm: the positive curvature of magnetic moment from $\mu_{0}H$ = 1.5 T to $\mu_{0}H=$ 5 T becomes negative for NP sizes below 10 nm.

In order to check whether saturation could be reached for higher $\mu_{0}H$-fields, M(H) up to $\mu_{0}H$ = 9 T has been measured for the bulk GdCu${}_{2}$ alloy (see inset of Figure 5a). As it can be noticed, not even at $\mu_{0}H$ = 9 T saturation can be reached. This is in good agreement with what has been reported in [39] for a GdCu${}_{2}$ single crystal, where the saturation was reached at $\mu_{0}H$ = 12 T. Accordingly, the magnetic saturation for the NPs should be found at even higher fields.

If we inspect in more detail the $M(\mu_{0}H)$ curves for both SSG NPs (〈D〉≤10 nm), a value of $M≅5\mu {}_{B}$ is reached at $\mu_{0}H$ = 5 T; *T* = 5 K, which is ∼70% of the theoretical saturation value *M* = $g{}_{J}\cdot J$ = 7 $\mu {}_{B}$ (*J* = 7/2, $g{}_{J}$ = 2). This is greater than the ∼55% value observed in TbCu${}_{2}$ NPs at the same magnetic field [21]. This finding indicates a lower anisotropy for GdCu${}_{2}$ NPs (no magnetocrystalline anisotropy) with respect to TbCu${}_{2}$ ones.

Table 3 gathers the values for the magnetic moment at 5 T, $M(\mu_{0}H=5$ T, T=5 K), coercive field $\mu_{0}H{}_{C}$ and remanent magnetic moment $M{}_{r}$. The left inset of Figure 5a shows a zoom-in of the central region of the hysteresis loops where both the coercive field and the remanent magnetic moment can be observed. Obviously, the bulk alloy (*t* = 0 h) is not shown as it does not display $\mu_{0}H{}_{C}$ due to its pure AF order. The increase of both $\mu_{0}H{}_{C}$ and $M{}_{r}$ with decreasing size, as indicated in Table 3, is in clear connection to the destruction of the AF state. In fact, there is a huge uprise of both magnitudes when crossing the 〈D〉≈ 18 nm (t= 1.75 h) limit, where the AF state still remains.

If we focus now on the behaviour of $\mu_{0}H{}_{C}$ (*T* = 5 K) vs. 〈D〉 (see Figure 5b), a maximum for $\mu_{0}H{}_{C}$ can be noticed for 〈D〉≃ 18 nm (*t* = 1.75 h milled NPs). The occurrence of a maximum is expected for FM systems, as size effects provoke the crossover from multi-domain to single-domain magnetic behaviour. However, this a novel fact for AF NP alloys, as the general trend is a continuous increase in $\mu_{0}H{}_{C}$ as size (Spin Glass phase) reduces (increases) [21,34]. This is connected to the idea that there is a specific NP size for which the strength of the SG phase is maximum, as it has been explained before. Further size reduction leads to a weakening of this phase, as the competing interactions among randomly-oriented magnetic moments are progressively destroyed.

Finally, considering the coexistence of FM and AF interactions in some of our samples, we have investigated the presence of Exchange Bias. The loops were measured after cooling down to both *T* = 5 K and *T* = 2 K in a presence of $\mu_{0}H$ = 5 T. However, our measurements (not shown here) indicate that no shift in the M(H) loop for any of the studied alloys. This absence of shift is not so surprising considering that the exchange anisotropy effect is weak when the interface of the core and the shell of the NPs presents atomic roughness [44]. This atomic roughness is a consequence of the crystalline microstrain.

#### 3.2.3. Dynamic Magnetic Susceptibility

**Figure 6:**
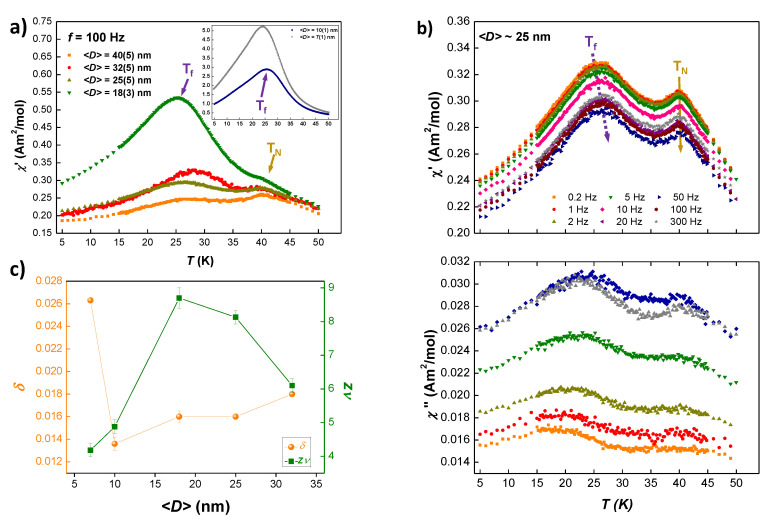
(**a**) Real $\chi^{\prime }\left(T,f\right)$ component of AC-susceptibility measured at *f* = 100 Hz for all of GdCu${}_{2}$-NPs. In the inset, NPs of 〈D〉= 10(1) nm and 〈D〉= 7(1) nm, where no trace of $T_{N}$ is found. (**b**) Real $\chi^{\prime }$ (**top**) and imaginary $\chi^{\prime \prime }$ (**bottom**) components of AC-susceptibility measured for GdCu${}_{2}$ 1.5 h milled at several frequencies. Arrows indicate the position of $T_{f}$ and $T_{N}$. (**c**) Evolution of δ and zν with mean particle size 〈D〉. A minimum (maximum) for δ-parameter (zν) is reached for 〈D〉 = 18 nm i.e., t= 1.75 h.

Dynamic magnetic susceptibility is a powerful technique that can provide valuable information on the Spin Glass dynamics [35]. Figure 6a shows the real $\chi^{\prime }\left(T\right)$ component of the AC-susceptibility [$\chi {}_{AC}\left(T,f\right)$] measured at *f* = 100 Hz for 〈D〉≈ 40, 32, 25, 18, 10 and 7 nm (*t* = 0.5, 1, 1.5, 1.75, 2 and 5 h respectively) GdCu${}_{2}$ NPs. A clean signal with two peaks for 〈D〉≥ 18 nm (i.e., milling times up to *t* = 1.75 h) can be observed (marked with arrows). These curves resemble the shape of the ZFC-$M{}_{DC}\left(T\right)$ measurements. The high temperature peak located at around 40 K (marked with a dark yellow arrow) corresponds to the Néel transition. As expected in a second-order phase transition, no shift with the frequency for this $T_{N}$ is observed (see Figure 6b top for 〈D〉≈ 25 nm). The low temperature peak located at around T= 25 K (marked with a purple arrow) corresponds to the transition to a Spin Glass-like state. The intensity of this freezing transition increases when the NP size is reduced. This rise in intensity is especially remarkable for 〈D〉≤ 10 nm (t≥ 2 h), as seen in the inset of Figure 6a. The $T_{f}$ shows a right-shift in temperature and a progressive reduction in intensity when increasing the frequency (see Figure 6b top panel), which follows the general trend for SG [35].

On the other hand, the imaginary $\chi^{\prime \prime }\left(T,f\right)$ (see Figure 6b bottom for 〈D〉≈ 25 nm) follows the features of the real component [45]. The appearance of a second peak located around T∼ 40 K may be connected to the existence of some FM interactions, as a pure AF alloy should not display a shoulder in $\chi^{\prime \prime }\left(T,f\right)$ [21].

The dynamics of the magnetic moments nearby the freezing transition can be quantified by focusing on different critical exponents [21,22,46]. The obtained values for these are gathered in Table 4.

First, the δ parameter, defined as $\delta =\ln(T{}_{f})/\log_{10}\left(2\pi f\right)+k$, where *k* is a constant, analyses the shift in temperature for $T_{f}$. This temperature shift is related to the freezing dynamics, which depends on the interactions (RKKY and/or dipolar) established among the moments. As it can be seen in Table 4, the values obtained for these GdCu${}_{2}$ NPs are below the upper limit of δ=0.06 found for SSG systems [47]. Instead, they are closer to the ones typically reported in other intermetallic SSG systems [21]. At the same time, these values are much higher than those typically attributed for the canonical SG state systems [(0.002–0.004) [48]], where the concentration of the magnetic impurities is very diluted [35].

Second, the freezing transition can also be characterised by a dynamic critical exponent zν, assuming that the spins participating in the freezing follow a critical slowing down. When the characteristic temperature of freezing, $T_{f}$, is reached, the transition relaxation diverges. The relaxation time for the decay of the fluctuations τ is related to the spin correlation length ξ according to $\tau \propto \xi^{z}$ [35,49]. The expression that relates the measuring time $\tau {}_{m}$ (=1/f) with the dynamic exponent zν is the following:

(2) $$\tau {}_{m}=\tau_{0}{\left(\frac{T_{f}-T{}_{f,0}}{T{}_{f,0}}\right)}^{z\nu }$$

where $T_{f,0}$ corresponds to the value of $T_{f}$ in the limit of f→ 0 and $\tau_{0}$ takes into account the relaxation time of individual particles when f→0. The value of $\tau_{0}$ can vary depending on the concentration of the disordered materials. In a seminal study it was concluded that for canonical SG $\tau_{0}$ = 10${}^{-13}$ s [50] and a huge number of studies were based on this assumption. More recently [10${}^{-8}$–10${}^{-12}$] s have been proposed for other canonical SG [51]. The relevant point is that, when NPs are involved, the value of $\tau_{0}$ tends to increase substantially as a consequence of the coupling of thousand of moments within the particle, and $\tau_{0}$ can reach values around 10${}^{-6}$ s [45]. The faster this relaxation takes place, the more disordered spins interact within the alloy. In our case, the best combination of zν, $T_{f,0}$ and $\tau_{0}$ are inserted in Table 4.

The values of $\tau_{0}$ calculated for our GdCu${}_{2}$ NPs lie within the aforementioned range for SSG systems, with a faster relaxation process with respect to other RCu${}_{2}$ SG NPs [21]. Regarding the values of the dynamic critical exponent zν, they are within the reported range for SG systems (4<zν<12 as in [51]). Here, it is worth noting how the values of zν drop when t≥ 2 h, following the suppression of the AF interactions explained before. Concerning $T_{f,0}$, it can be seen that they are smaller than the measured $T_{f}$, as the true SG phase is reached only when f→0 [48]. Once again, a reduction of this value is detected when the AF order is completely destroyed. These values of $T_{f,0}$ show only a 2 K deviation with respect to the ones extrapolated from the AT-line fitting discussed in the static magnetisation, which indicates a good agreement between both static and dynamic characterisations [37].

It is worth paying attention to the evolution of δ and zν parameters with the NP size (see Figure 6c). As GdCu${}_{2}$ NPs reduce in size, δ reduces as well, whereas zν shows a tendency to increase. This clearly points to increasing interactions among NPs, totally consistent with the M${}_{DC}\left(T\right)$ results. However, for 〈D〉≈18 nm (t= 1.75 h), a minimum (maximum) for δ (zν) is reached, followed then by a change in this tendency for smaller sizes. This change indicates a reduction in the interactions among magnetic moments. The finding of this extreme in both dynamic exponents is in good agreement with the extreme found in the IA or FP $M{}_{DC}$ measurements. It illustrates that when the AF order vanishes, the intensity of the interactions decreases, which is reflected in the sudden change of the critical exponents.

Third, the temperature dependence of the characteristic relaxation time for spin glass systems can be obtained from a dynamic scaling of the imaginary $\chi^{\prime \prime }\left(T,f\right)$ [45,49]. If we consider $\varepsilon =\left(T-T{}_{f,0}\right)/T{}_{f,0}$ we can obtain the order parameter critical exponent β as:

(3) $$\chi^{\prime \prime }\left(f/2\pi ,T\right)=\varepsilon^{\beta }{\left[ℑ\left(f/2\pi \right)\varepsilon \right]}^{z\nu }$$

The obtained β values for our GdCu${}_{2}$ NPs are within the expected range of [0.6–0.8] [52] for SG systems and the one of β= 0.75 (0.25) found for Heisenberg-like SG 3D systems with low anisotropy [53].

**Figure 7:**
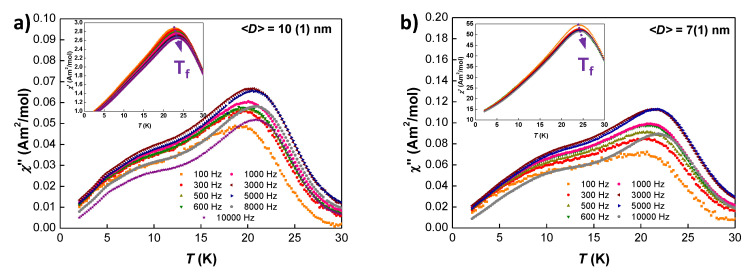
Imaginary $\chi^{\prime \prime }\left(T,f\right)$ component of the AC-susceptibility measured for (**a**) 〈D〉=10(1) nm and (**b**) 〈D〉 = 7(1) nm GdCu${}_{2}$ NPs. The insets show the real $\chi^{\prime }\left(T,f\right)$ component, where the position of $T_{f}$ is marked by a purple arrow.

Finally, the imaginary $\chi^{\prime \prime }\left(T,f\right)$ curves for pure SSG NPs (t≤ 10 nm) have revealed relevant information about the different freezing processes that undergo the magnetic moments located within the NP core and shell. Figure 7 shows the $\chi^{\prime \prime }\left(T\right)$ measured at f= 100 Hz for 〈D〉=10(1) nm and 〈D〉=7(1) nm. Here, $\chi^{\prime \prime }\left(T,f\right)$ component presents two extra shoulders in the low temperature regime, which are not observed in the real part. This finding of a doubled-peak signature in $\chi^{\prime \prime }\left(T,f\right)$ for SSG NPs can be associated with a two-step freezing process, as has also been observed in Fe/$\gamma -Fe_{2}O_{3}$ core-shell SSG nanoparticles [47,54]. The aforementioned process involves, first, the freezing of the magnetic moments located within the core, giving rise to the shoulder found at T∼13 K; and second, the freezing of the magnetic moments at the shell, giving rise to the shoulder found at T∼ 7 K. The ratio shell/core moments for these small NPs is around $\frac{N{}_{S}}{N{}_{C}}∼\frac{6<a>}{D}∼0.4$. Therefore, the magnetic response of both shell and core magnetic moments is relevant enough to leave a trace in $\chi^{\prime \prime }\left(T,f\right)$.

## 4. Conclusions

The evolution of the magnetic properties with respect to the size reduction and the microstrain has been analysed thanks to a series of six GdCu${}_{2}$ nanometric alloys. The macroscopic magnetic characterisation has been carried out from both static and dynamic magnetic points of view. First, the analysis has revealed how the AF bulk state gets progressively destroyed with milling time, whereas a disordered magnetic contribution emerges due to both size reduction and increasing microstrain. These AF interactions mainly remain within the core of the NPs, according to the scenario suggested by both $MT_{f}{}_{DC}\left(H,T\right)$ and $\chi {}_{AC}\left(T,f\right)$.

Second, the analysis of GdCu${}_{2}$ NPs has evidenced the existence of a threshold that separates two different magnetic states: The one for 〈D〉≥ 18 nm (t≤ 1.75 h), where the AF interactions coexist together with the frustrated and disordered magnetic moments (Spin Glass) located at the shell, leading to a Superantiferromagnetic state; and the one for 〈D〉≤ 10 nm (t≥ 2 h), in which the AF interactions are destroyed and a Super Spin Glass state is formed, where all the magnetic moments are frustrated. Concerning the later SSG state, we have been able to observe traces of a separate freezing for the core and the shell at 〈D〉 = 10(1) nm and 〈D〉 = 7(1) nm NPs. This two-step freezing is a non common observation. Hence, the selection of the the low anisotropy of $Gd^{3+}$ (L=0) has allowed us to unveil the subtle magnetic coupling/uncoupling process in the NPs. Understanding the later is conducive for many applications, such as magnetic recording and magnetic hyperthermia.

Third, the evolution of the magnetic disorder with the nanoparticle size is specially striking, being maximised for NPs of 〈D〉 = 25(5) nm, where the AF interactions are still present. This finding is explained by means of the two sources of magnetic disorder frustration that are established for that NP size: a first that comes from the magnetically disordered shell moments (always present within the nanoscale), and a second one, which results from the competition between the FM-AF RKKY interactions (only present for 〈D〉≥ 18 nm). Both analyses of static M(H) and dynamic ($\chi_{AC}$) measurements, together with the two novel quantities presented in this work, the IA and the FP, have been used to quantify the stability of this SG state. The later IA and FP quantities provide new simple tools that can help the overall interpretation of the influence of the structure (size and microstrain) on the physical properties of nanomagnets.

## Figures and Tables

**Table 1:** Orthorhombic mean lattice parameters (*a*,*b* and *c*); relative change in the unit size volume cell with respect to the unit cell in bulk alloy (ΔV), size 〈D〉, microstrain η and Bragg factor $R_{B}$ of nanoparticles at different milling times (*t*). Bragg factors (R${}_{B}$) close to 10% ensure the reliability of our refinements.

*t* (h)	*a* (Å)	*b* (Å)	*c* (Å)	ΔV (%)	〈D〉 (nm)	η (%)	$R_{B}$ (%)
0.5	4.329(4)	6.886(1)	7.342(1)	0.6	40(5)	0.14(5)	10.2
1	4.332(1)	6.903(2)	7.349(1)	0.3	32(5)	0.13(5)	11.5
1.5	4.326(1)	6.895(1)	7.340(2)	0.9	25(5)	0.17(5)	12.9
1.75	4.328(2)	6.903(2)	7.343(3)	0.1	18(3)	0.23(5)	6.7
2	4.314(3)	6.878(1)	7.304(2)	1.1	10(1)	0.47(9)	3.2
5	4.314(4)	6.887(1)	7.317(3)	0.8	7(1)	0.95(2)	2.0

**Table 2:** Irreversibility temperature associated with the freezing (SG) transition measured at 0.25 mT, T${}_{f}(H=$ 0.25 mT), $T{}_{irrev}$ obtained from AT line extrapolation described in Equation (1), paramagnetic Curie temperature $\theta {}_{P}$ and effective magnetic moment $\mu_{eff}$ obtained from Curie-Weiss fitting of FC measurements taken at $\mu_{0}H$ = 0.1 T for the different GdCu${}_{2}$ NPs.

〈D〉 (nm)	$T{}_{f}$$(\mu_{0}H=0.25$ mT) (K)	*T*${}_{\mathrm{irrev}}$$\left(\mu_{0}H=0\right)$ (K)	$\theta {}_{P}$ (K)	$\mu_{\mathrm{eff}}\left(\frac{\mu {}_{B}}{\mathrm{Gd}\mathrm{at}}\right)$
40	32	–	8.16(2)	8.762(1)
32	31	26.4(3)	8.03(2)	8.876(1)
25	30(1)	27.4(4)	8.17(7)	8.763(1)
18	28(1)	24.0(3)	10.6(3)	8.697(1)
10	24.2(5)	21.7(2)	21.7(3)	8.452(1)
7	24.3(5)	21.6(2)	23.0(2)	8.703(1)

**Table 3:** Magnetic moment at $\mu_{0}H$ = 5 T M(5T), coercive field $\mu_{0}H{}_{C}$ and remanent magnetic moment $M_{r}$ for GdCu${}_{2}$ NPs measured at *T* = 5 K.

〈D〉 (nm)	M(5T) $\left(\mu {}_{B}/\mathrm{Gd}\right)$	$\mu_{0}H{}_{C}$ (mT)	$M_{r}\;(10^{-3}$$\mu {}_{B}/\mathrm{Gd}$)
40	2.33(2)	10.0(5)	5.83(2)
32	2.33(2)	15.0(5)	7.25(2)
25	2.34(1)	15.0(5)	6.58(2)
18	2.72(3)	20.0(5)	20.8(2)
10	4.61(2)	10.0(5)	114(1)
7	4.86(2)	5.0(5)	126(2)

**Table 4:** δ-shift parameter, relaxation time $\tau_{0}$ of individual particles for f→0, freezing transition temperature $T_{f}$, critical exponent zν and β critical exponent for all of the GdCu${}_{2}$ NPs.

〈D〉 (nm)	δ	$\tau_{0}$ (s)	$T_{f,0}$ (K)	zν	β
40	0.0280(2)	10${}^{-8}$	24.3(4)	7.7(3)	0.70(5)
32	0.0180(1)	10${}^{-8}$	26.5(2)	6.1(2)	0.80(5)
25	0.0160(1)	10${}^{-13}$	25.14(2)	8.1(2)	0.80(5)
18	0.0160(5)	10${}^{-13}$	24.13(2)	8.7(3)	0.90(5)
10	0.0136(6)	10${}^{-11}$	22.58(4)	4.9(2)	0.60(5)
7	0.0263(1)	10${}^{-8}$	23.20(4)	4.2(2)	0.50(5)

## Abbreviations

The following abbreviations are used in this manuscript:

AF	Antiferromagnetic
CEF	Crystal Electric Field
CGS	Cluster Spin Glass
EB	Exchange Bias
FC	Field Cooling
FM	Ferromagnetic
FP	Spin Glass Sharpness
IA	Irreversibility Area
MNPs	Magnetic Nanoparticles
$\mu_{0}H{}_{C}$	Coercitive Field
NP	Nanoparticle
R	Rare Earth
SAF	Superantiferromagnetism
SG	Spin Glass
SSG	Super Spin Glass
ZFC	Zero Field Cooling
